# Photoelectrochemical oxidation of organic substrates in organic media

**DOI:** 10.1038/s41467-017-00420-y

**Published:** 2017-08-30

**Authors:** Tengfei Li, Takahito Kasahara, Jingfu He, Kevan E. Dettelbach, Glenn M. Sammis, Curtis P. Berlinguette

**Affiliations:** 1 Department of Chemistry, The University of British Columbia, 2036 Main Mall, Vancouver, Canada BC V6T 1Z1; 2 Department of Chemical & Biological Engineering, The University of British Columbia, 2036 Main Mall, Vancouver, Canada BC V6T 1Z1; 3 Present Address: Stewart Blusson Quantum Matter Institute and Department of Chemical ‖ Biological Engineering, The University of British Columbia, 2036 Main Mall, Vancouver, Canada BC V6T 1Z1

## Abstract

There is a global effort to convert sunlight into fuels by photoelectrochemically splitting water to form hydrogen fuels, but the dioxygen byproduct bears little economic value. This raises the important question of whether higher value commodities can be produced instead of dioxygen. We report here photoelectrochemistry at a BiVO_4_ photoanode involving the oxidation of substrates in organic media. The use of MeCN instead of water enables a broader set of chemical transformations to be performed (e.g., alcohol oxidation and C-H activation/oxidation), while suppressing photocorrosion of BiVO_4_ that otherwise occurs readily in water, and sunlight reduces the electrical energy required to drive organic transformations by 60%. These collective results demonstrate the utility of using photoelectrochemical cells to mediate organic transformations that otherwise require expensive and toxic reagents or catalysts.

## Abstract (web summary)

Photoelectrochemical water splitting is a promising method for H_2_ fuel production, but the O_2_ by-product generated has little economic value. Here, Berlinguette and colleagues demonstrate that BiVO_4_ photoanodes immersed in organic media can instead perform valuable alcohol oxidation and C–H functionalization reactions.

## Introduction

Interfacial photoelectrochemistry (PEC) at a semiconductor-liquid junction enables the direct conversion of light into chemical energy^1–3^. The highly energetic electrons and holes that are created through the absorption of visible light by semiconducting electrodes are capable of driving the energetically demanding conversion of water into oxygen and hydrogen fuels^4–6^. While there has been a large global effort to reduce the relatively large energy losses associated with producing oxygen at the anode, strikingly few photoanodes are known to be capable of both absorbing incident sunlight and mediating efficient and sustained water oxidation catalysis^7–11^.

The technical challenges of using a photoanode to form dioxygen that has little economic value provides the imperative to explore alternative reactions that generate higher value products with lower energy input. Choi and coworkers recently demonstrated that the photoelectrochemical oxidation of 5-hydroxymethylfurfural into 2,5-furandicarboxylic acid could be realized with a BiVO_4_ photoanode in the presence of a redox mediator^12^. A key challenge of this distinctive approach, however, is that the aqueous medium limits the scope of chemistry available and induces high rates of BiVO_4_ photocorrosion^13, 14^. While photocorrosion in H_2_O can be suppressed by a protective (electrocatalytic) layer (e.g., CoO_x_, NiO_x_, and FeO_x_)^14–16^, these layers tend to suppress the oxidation of organic compounds in favor of competitive oxygen evolution^12, 17^.

**Fig. 1:**
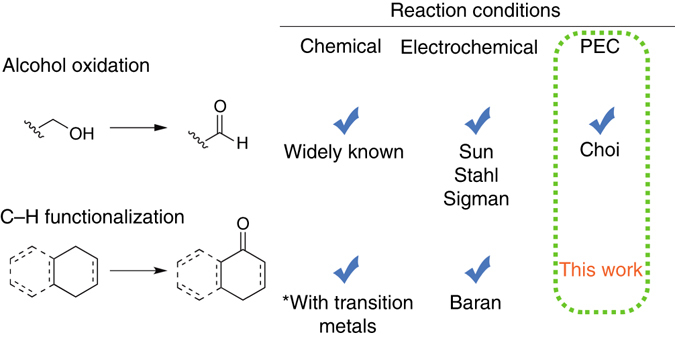
Summary of reaction conditions for alcohol oxidation and C-H functionalization. Three types of reaction conditions (chemical oxidation, EC oxidation at an electrode^20–22, 26^, and photoelectrochemical oxidation^12^) that have been used for alcohol oxidation and C–H functionalization are shown. We report here C–H functionalization and oxidation by PEC

We therefore set out to explore conditions where the photoelectrochemically-driven oxidation of organic compounds in non-aqueous media could be achieved. Two classes of well known organic transformations were chosen to test this concept: alcohol oxidation and the more challenging C–H functionalization/oxidation (Fig. 1). The oxidation of alcohols can be readily achieved using chemical oxidizing agents that include transition metals, hypervalent iodine reagents, and activated sulfur transformations^18, 19^. Some of these alcohol oxidation reactions have also been conducted using an electrode^20–22^, photo-oxidized dyes immobilized on TiO_2_^23^, and, as mentioned above, a photoanode^12^. The activation and subsequent functionalization of C–H bonds is a greater challenge^24^. The respective transformation of olefin and alkylbenzene compounds into α,β-unsaturated and aryl ketones, for example, usually requires toxic reagents (e.g., CrO_3_ and SeO_2_) and/or expensive metal catalysts (e.g., Rh and Pd)^24, 25^, but allylic C–H oxidation can also be driven electrochemically at a carbon electrode^26, 27^. Note that the electrochemical (EC) study by Baran and coworkers was performed in non-aqueous media, thereby enabling them to oxidize a large set of substrates with reasonable yields (40–90%)^26^.

We report here the use of a PEC cell to mediate organic oxidation reactions using a photoanode immersed in organic media. The photoelectrochemical oxidation of benzyl alcohol, cyclohexene, and tetralin into their corresponding carbonyl compounds were demonstrated in a PEC cell containing a BiVO_4_ photoanode immersed in MeCN containing appropriate reaction mediators (e.g., electrolyte, base, and electron transfer reagent). Product formation can be achieved using a 60% reduction in the applied voltage compared to a purely EC oxidation. Furthermore, no significant degradation of the photoanode was observed.

## Results

### Photoelectrochemical stability of BiVO_4_ in non-aqueous media

**Fig. 2:**
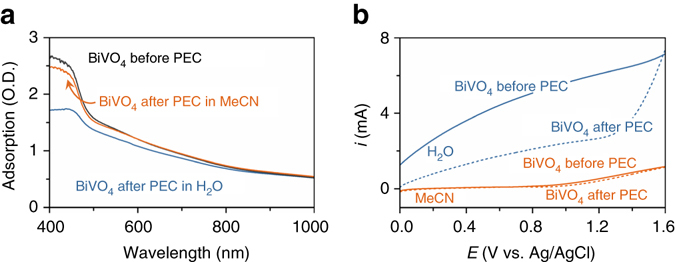
Demonstration of lower BiVO_4_ photocorrosion in MeCN than in H_2_O. **a** UV-Vis absorption spectra of BiVO_4_ photoanodes before (*black*) and after 96 h of PEC in H_2_O (*blue*) or MeCN (*orange*). **b** Photocurrents of BiVO_4_ photoanodes before (*solid line*) and after (*dashed line*) 96 h of PEC electrolysis in H_2_O (*blue*) or MeCN (*orange*)

The BiVO_4_ photoanodes used in this study were synthesized in accordance with documented procedures^28^. Appropriate stoichiometric amounts of bismuth nitrate hexahydrate and vanadyl acetylacetonate precursors dissolved in a solution of acetic acid and acetylacetone were spin-coated onto fluorine-doped tin oxide (FTO), annealed at 500 °C, and then subjected to ultraviolet (UV) irradiation (*λ*_max_ ~185 and 254 nm, flux ~10 mW cm^−2^) in air for 20 h. This UV radiation step renders higher activity for aqueous PEC^28, 29^. The UV-Vis absorption spectra, surface morphology, and powder X-ray diffractograms of the films are consistent with previous descriptions of BiVO_4_ prepared by this protocol (Fig. 2, Supplementary Figs. 1 and 2)^10, 28^.

The photoelectrochemical stability of BiVO_4_ in aqueous and non-aqueous media was tested by measuring the absorptivity changes before and after 96 h of illumination under an applied potential. The photoanodes were immersed in 25 mL H_2_O containing 0.1 M NaHCO_3_ or 25 mL MeCN with 0.1 M LiClO_4_ and exposed to simulated AM1.5 G sunlight for 24 h and an applied potential of 0.1 or 1.6 V, respectively (all potentials reported herein are referenced against Ag/AgCl). The anodes were then left in solution in the dark for 24 h at open-circuit prior to a subsequent 24 h of PEC electrolysis at these same potentials. UV-Vis absorption spectra of the photoanodes recorded after four successive cycles in H_2_O (i.e. ~96 h of cumulative exposure to PEC conditions) showed a much more significant reduction in intensity over the 400–600 nm range than those in MeCN (Fig. 2a). This reduced absorptivity is consistent with a decrease in BiVO_4_ film thickness, while the nominal changes in absorptivity of the photoanode in MeCN after PEC indicate insignificant photocorrosion. The solvent-dictated differences in photoanode stability were corroborated by photocurrent measurements of BiVO_4_ before and after PEC in both solvents (Fig. 2b): The anode tested in H_2_O exhibited a striking drop in photocurrent after 96 h of PEC electrolysis, whereas the photocurrents recorded in MeCN did not change. The photoanodes tested in MeCN with increasing amounts of water also showed an increasing propensity for photocorrosion (Supplementary Table 1). The accelerated photocorrosion of BiVO_4_ in H_2_O was confirmed by scanning electron microscope (SEM) images of photoanodes before and after 96 h of PEC. Full coverage of FTO by spherical grains of BiVO_4_ (Supplementary Fig. 2) are retained for the photoanodes subjected to 96 h of PEC electrolysis in MeCN, with visible FTO providing evidence for less uniform coverage in films exposed to aqueous PEC. This morphology change during PEC electrolysis in H_2_O is widely known^14, 16^. While organic solvents increase the resistance of the PEC cell (~10–20 Ω with MeCN *c.f*. <10 Ω using water), merely ~30 mV in additional voltage is required at the low currents (~3 mA) relevant to this study. This additional voltage represents a modest energy penalty for the much higher photoanode stability.

### Photoelectrochemical alcohol oxidation

Having validated the photostability of BiVO_4_ in MeCN, we tested the oxidation of organic substrates using a PEC cell. All experiments were carried out in MeCN containing LiClO_4_ electrolyte. Other organic solvents (e.g. acetone and CH_3_NO_2_) and electrolytes (e.g. Bu_4_NClO_4_ and Bu_4_NPF_6_) were tested but did not yield any discernible benefit in photocurrent or product yield. MeCN was utilized as the solvent because because the boiling point (82 °C) is sufficiently high to minimize evaporation during electrolysis and sufficiently low to circumvent interfering with the gas chromatography-mass spectrometry **(**GC-MS) analysis. LiClO_4_ was selected on the basis of it being a cheap electrolyte that is soluble in organic media.

**Fig. 3:**
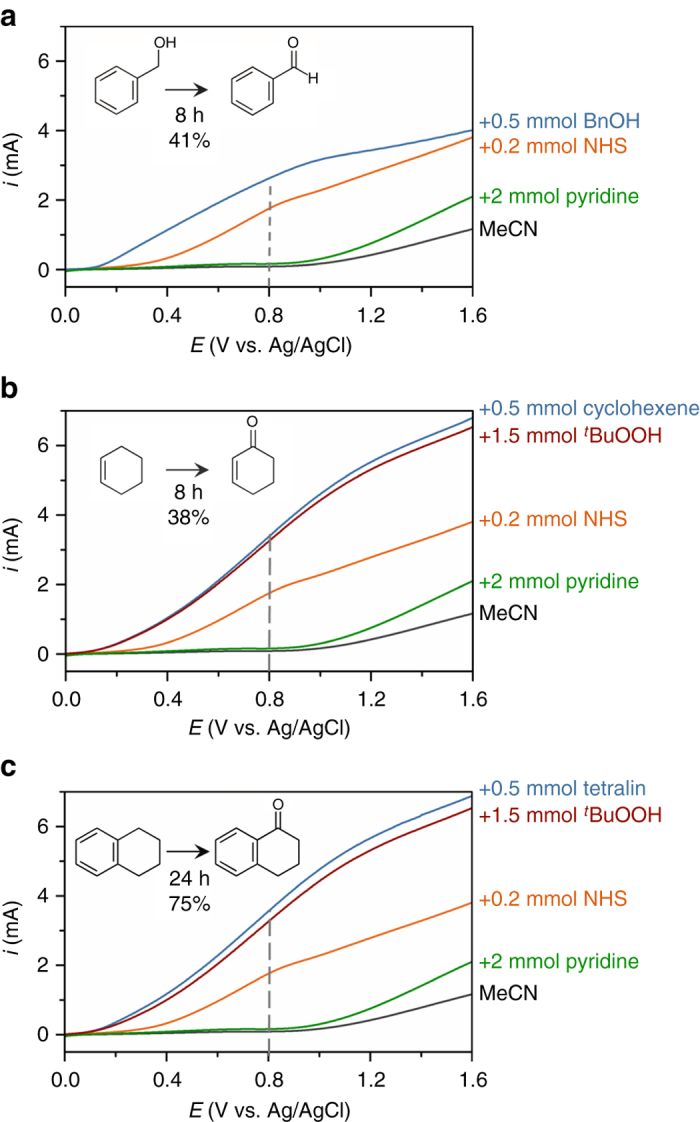
LSV curves of PEC organic oxidation profiles. Photocurrents correspond to PEC oxidations of **a** benzyl alcohol to benzaldehyde, **b** cyclohexene to cyclohexanone, and **c** tetralin to 1-tetralone using a BiVO_4_ photoanode immersed in 25 mL MeCN containing 0.1 M LiClO_4_ and subjected to AM1.5 G light (scan rate = 10 mV s^−1^). Photocurrent profiles correspond to the solvent and electrolyte solution (*black*) after the successive addition of 2 mmol pyridine (*green*), 0.2 mmol NHS (*orange*), 1.5 mmol of ^*t*^BuOOH (*red*) and 0.5 mmol of the respective substrate (*blue*). The reaction yields determined by GC-MS are indicated. The *dashed line* indicates the NHS^−^ → NHS^●^ oxidation; this potential was used as the applied voltage, *V*_app_, for PEC electrolysis experiments

Linear sweep voltammetry (LSV) was measured under AM1.5 G light prior to performing PEC electrolysis (Fig. 3). Photocurrent profiles recorded on the BiVO_4_ working electrode in 25 mL of MeCN containing 0.1 M LiClO_4_, a glassy carbon counter electrode (CE), and an Ag/AgCl reference electrode (RE), generated nominal current below ca. 1 V. For each of the organic reactions in this study, pyridine served as the requisite base to accommodate deprotonation of *N*-hydroxysuccinimide (NHS), a soluble, transparent, and electrochemically active species that proved effective at mediating hole transfer from the electrode to the organic substrate. Baseline photocurrent profiles were measured prior to addition of 0.5 mmol of substrate: 4 equiv. pyridine (162 µL; 2 mmol) caused a small but detectable change in LSV photocurrent below 1 V (Fig. 3), while the subsequent addition of 40 mol% NHS (23 mg; 0.2 mmol) caused a more dramatic rise in current. The shoulder at 0.8 V (indicated by the vertical dashed lines in Fig. 3) is assigned to the oxidation of NHS^−^ to electrochemically-active NHS^●^^30^. All organic oxidation reactions in this report were therefore carried out under an applied voltage of 0.8 V (denoted *V*_app_), which is sufficiently low to avoid solvent and pyridine oxidation yet sufficiently positive to generate the NHS^●^ species required for oxidation of the organic substrates. The BiVO_4_ photoanodes after 96 h of PEC with substrate exhibit minor differences in absorptivity and LSV photocurrents consistent with negligible photocorrosion (Supplementary Figs. 2 and 3), but not to a degree that compromises these experiments.

The facile oxidation of benzyl alcohol to benzaldehyde was selected as a proof-of-concept experiment because alcohol oxidation was previously shown to proceed at a BiVO_4_ photoanode in H_2_O^12^. The photocurrent increased after the addition of 0.5 mmol benzyl alcohol to the PEC cell described above (Fig. 3a). This increase is assumed to be due to the consumption of NHS^●^ (which oxidizes benzyl alcohol) driving a faster NHS^−^ oxidation. The oxidation of benzyl alcohol into benzaldehyde was confirmed by GC-MS, with a reaction yield of 41% after 8 h of PEC electrolysis (Supplementary Fig. 4). While further optimization is still needed to make the yield of benzaldehyde competitive with the best available synthetic methods, the result demonstrates the viability of performing PEC in a non-aqueous medium. These experiments also demonstrate that NHS can act as a useful redox mediator in tandem with a BiVO_4_ photoanode.

### Photoelectrochemical C-H oxidation

With evidence of a successful photoelectrochemical oxidation in non-aqueous media, we next explored the more synthetically challenging oxidation of cyclohexene to cyclohexenone (Fig. 3b) and tetralin to 1-tetralone (Fig. 3c). In addition to using NHS as a redox mediator, an external oxygen source, *tert*-butyl hydroperoxide (^*t*^BuOOH), is used to form the requisite ^*t*^Bu peroxide-substrate adduct^26^. (Supplementary Fig. 8) Prior to addition of 0.5 mmol of substrate, 3 equiv. of ^*t*^BuOOH were shown to produce larger photocurrents than what was measured for pyridine, presumably due to the oxidation of ^*t*^BuOOH. The PEC electrolysis experiments afforded cyclohexanone from cyclohexene (38% yield after 8 h, Supplementary Fig. 5) and 1-tetralone from tetralin (75% yield after 24 h; Supplementary Fig. 6a). The conversions were quantified by GC-MS to confirm 93% conversion of tetralin to 1-tetralone, which is corroborated with the coulombic charge passed through the PEC cell (Supplementary Figs. 6b and 7).

The PEC oxidations of each of the compounds appear to be mediated by NHS. In each of these schemes, a quasi-static equilibrium of photo-generated electron and hole populations leads to the separation of the BiVO_4_ Fermi level (*E*_F_) into quasi-Fermi levels for electrons (*E*_F,n_) and holes (*E*_F,p_)^3^. The difference between these two quasi-Fermi levels represents the photovoltage (*V*_ph_), which serves to reduce the applied voltage (*V*_app_) required to drive the reaction. The interfacial chemistry is then driven by the oxidation of deprotonated NHS to form NHS^●^, which reacts with the substrate. (Control experiments ruled out a meaningful rate of oxidation without NHS; the replacement of NHS with *N*-methoxysuccinimide, which is not able to generate a radical, also did not show reactivity.) In the case of tetralin (a conjectured mechanism for tetralin photo-oxidation is provided in Supplementary Fig. 8), NHS^●^ abstracts a hydrogen atom from tetralin to ostensibly form a stable benzylic radical species that ultimately leads to a peroxide intermediate that eliminates ^*t*^BuOH to yield 1-tetralone. The reductive chemistry at the cathode is assumed to involve pyridinium. This proposal is in line with known chemistry^26^, but experiments are underway to validate the proposed reaction steps and energetic profiles for each of these reactions.

### Voltage reduction provided by PEC cell

**Fig. 4:**
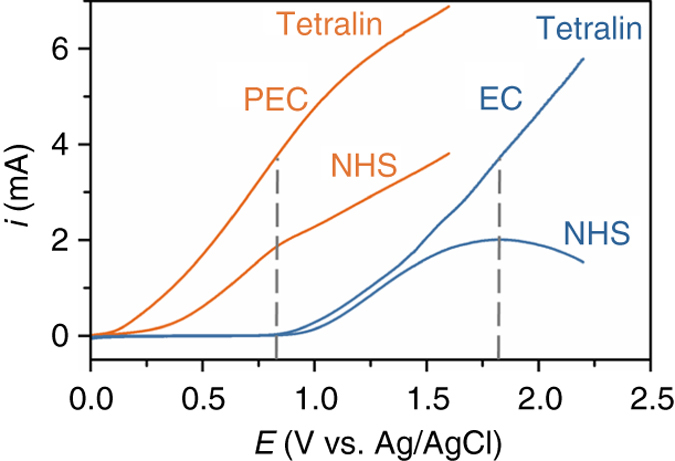
Contrasting the photoelectrochemical and EC oxidation processes. The NHS-mediated oxidation of tetralin in a PEC cell (*orange*) is cathodically shifted by 1 V compared to an EC cell (EC; *blue*). The (photo)currents measured after adding pyridine and NHS to MeCN are denoted NHS, while the (photo)currents measured after adding ^*t*^BuOOH and tetralin are denoted tetralin. The shoulder at 0.8 V and peak at 1.8 V (*dashed lines*) are attributed to the oxidation of NHS^−^ to NHS^●^ under PEC and EC conditions, respectively

We also quantified the electrical energy savings provided by AM1.5 G light for the oxidation of tetralin in the PEC cell vs. an EC cell with a glassy carbon electrode (Fig. 4). The oxidation of NHS^**−**^ to NHS^●^ was measured at 0.8 V and 1.8 V for the PEC and EC cell, respectively. These potentials each produced currents of ~3.8 mA and thus similar 75% yields of 1-tetralone after 24 h of (photo)electrolysis (Supplementary Fig. 7). This comparison indicates that illumination of the PEC cell enables *V*_app_ to be reduced by 1 V compared to that of the EC cell corresponding to a 60% reduction in energy savings.

The solar-to-electricity efficiency (*η*) of traditional PEC water oxidation can be calculated by the following relationship: *η* = J*(1.23 V−*V*_app_)/*P*_in_, where *J* = photocurrent density, 1.23 V = E^o^ (O_2_/H_2_O), *V*_app_ = applied potential in the PEC cell, and *P*_in_ = power density of illumination (100 mW cm^−2^ in this study). State-of-the-art BiVO_4_ photoanodes that mediate PEC water oxidation at a *V*_app_ of 0.6 V generate *J* = 2.73 mA cm^−2^ and therefore yield *η* = 1.7%. In our experiments in MeCN, the PEC cell was driven at 0.8 V to produce *J* = 3.8 mA/3 cm^2^ = 1.3 mA cm^−2^. Our EC cell required 1.8 V to yield the same *J* value of 1.3 mA cm^−2^, and thus we assumed 1.8 V to effectively be the pseudo standard potential of NHS. Given that our illuminated photoanode area was 3 cm^2^ and our light intensity was 100 mW cm^−2^, the *η* of our cell is therefore 1.3%. This analysis shows that our PEC organic oxidation system offers higher-valued organic products at approximately the same energy efficiency as traditional PEC water oxidation.

In summary, we have demonstrated that illumination of BiVO_4_ immersed in organic media drives both alcohol oxidation and C-H activation/oxidation. The use of light can reduce the applied voltage of an EC oxidation process by 1 V. The use of organic media in place of water affords markedly higher semiconductor photostability, thereby enabling sustained photochemical conversion over at least 1 day. This alternative PEC cell design highlights the potential to generate higher-value small molecules compared to traditional PEC cells.

## Methods

### Materials

Bismuth nitrate hexahydrate (98%), vanadyl acetylacetonate (98%), and tungsten hexachloride (99%) were purchased from Strem Chemicals and used as received. All other chemicals were purchased from commercial vendors (Acros Organics, Alfa Aesar, Fisher Scientific, or Sigma-Aldrich) and used as received. FTO substrates (TEC 15) were purchased from Hartford Glass Co. Glassy carbon electrodes were purchased from Alfa Aesar.

### Electrode preparation

BiVO_4_ photoanodes were prepared in accordance with previously reported procedures^10, 28^. Bismuth nitrate hexahydrate (0.346 g, 0.713 mmol), vanadyl acetylacetonate (0.176 g 0.663 mmol), and tungsten hexachloride (0.02 g, 0.05 mmol) were added to a 10 mL mixture of acetic acid and acetylacetone (1:8 *v/v*). The solution was sonicated for 15 min and then spin-coated onto a FTO substrate at 1000 rpm for 30 s. Each of the successive 16 coats was annealed at 500 °C for 10 min prior to a final 8 h annealing step at 500 °C. The BiVO_4_ samples were irradiated with UV light (Model #: GPH436T5VH, Atlantic Ultraviolet Co.; *λ*_max_ ~ 254 nm and 185 nm; flux ~10 mW cm^−2^ at 5.5 cm in our experiment) for 20 h. The geometric surface areas of all working electrodes (BiVO_4_ photoanodes and glassy carbon electrodes) in this study were 3 cm^2^.

### Photoelectrochemical and electrochemical oxidation

PEC and EC electrolysis were performed with a CHI 660D potentiostat in a three-electrode photoelectrochemical cell with a BiVO_4_ photoanode (for PEC experiments) or glassy carbon (electrochemistry experiments) as the working electrode, Ag/AgCl as the RE, and glassy carbon as the CE. PEC measurements and electrolysis were conducted under AM1.5 G simulated sunlight with an Oriel 94011A-ES solar simulator integrated with a 100-W xenon arc lamp and AM1.5 G filter. An infrared water filter was applied to prevent the solution from heating up during PEC electrolysis. Experiments were performed in 0.1M LiClO_4_ in 25 mL MeCN or 0.1M NaHCO_3_ in H_2_O (buffered to pH 7). Potentials were measured vs. Ag/AgCl, which was calibrated to 0.196 V vs. NHE in aqueous media. For the dark and photocurrent measurements, LSV was measured prior to performing electrolysis, and the scan rate was maintained at 10 mV s^−1^. For PEC and EC oxidations of organic substrates: pyridine (2 mmol, 4 equiv.), NHS (0.2 mmol, 40 mol%), ^*t*^BuOOH (70% in H_2_O, 1.5 mmol, 3 equiv. for cyclohexene and tetralin oxidations) and substrate (0.5 mmol, 1 equiv.) were added successively to a 0.1M LiClO_4_ solution in 25 mL MeCN. Control experiments were performed under the same condition, except that each of the said reagents was added in exclusivity. PEC/EC electrolysis was performed at room temperature, using a constant potential method (0.8 V and 1.8 V vs. Ag/AgCl for PEC and EC electrolysis, respectively) with magnetic stirring.

### Gas chromatography-mass spectrometry

Samples were taken during and after electrolysis and carvone was added as an internal standard. GC-MS measurements were run on an Agilent GC-MS instrument with a HP-5ms GC column and electron ionization ion source. A 1-μL aliquot was injected with an auto-sampler using split mode with a split ratio of 20:1, and carried by He gas at a flow rate of 1 mL min^−1^. The oven temperature started at 60 °C for 1 min, then ramped from 100 to 150 °C at 20 °C min^−1^ and from 150 to 250 °C at 40 °C min^−1^. Detection was not performed for the first 1.75 min of the run, or the solvent delay time. GC-MS peaks were identified using the NIST Mass Spectrometry Data Center database (all peak identifications exhibited a probability match of >90%) and compared with peaks in standard solutions. For quantitative analysis, a series of standard solutions with known concentrations of reactants and products were prepared and carvone was added as an internal standard. Standard solutions were run by GC-MS using the same method, and the relative peak areas of reactants and products were normalized to the internal standard peak. Linear calibration curves were plotted to quantify the concentrations of reactants and products.

### Physical methods

Absorptivity measurements of BiVO_4_ samples were collected with a PerkinElmer Lambda 35 UV-Vis spectrometer with a solid sample holder accessory. Baseline scans were recorded on clean FTO. X-ray diffraction characterization was conducted using a Bruker F8 Focus X-ray diffractometer. Data were collected between *2θ* angles of 5° and 90° with a step size of 0.04° and the step time was 0.6 s. Film morphologies were investigated using a Helios NanoLab 650 Focused Ion Beam SEM, with the accelerating voltage kept at 1 kV and the current at 50 pA for imaging.

### Data availability

The authors declare that the main data supporting the findings of this study are available within the article and its Supplementary Information files. Extra data are available from the corresponding author upon request.

## Electronic supplementary material

Supplementary Information
