# Supplementary material for: Photoelectrochemical oxidation of organic substrates in organic media
Source: Nat Commun. 2017 Aug 30;8:390. doi: 10.1038/s41467-017-00420-y (PMC5577226; doi:10.1038/s41467-017-00420-y)
Supplement: Supplementary file 1 — Supplementary Information [file 41467_2017_420_MOESM1_ESM.pdf]

Description of Supplementary Files

File Name: Supplementary Information
Description: Supplementary Figures and Supplementary Table

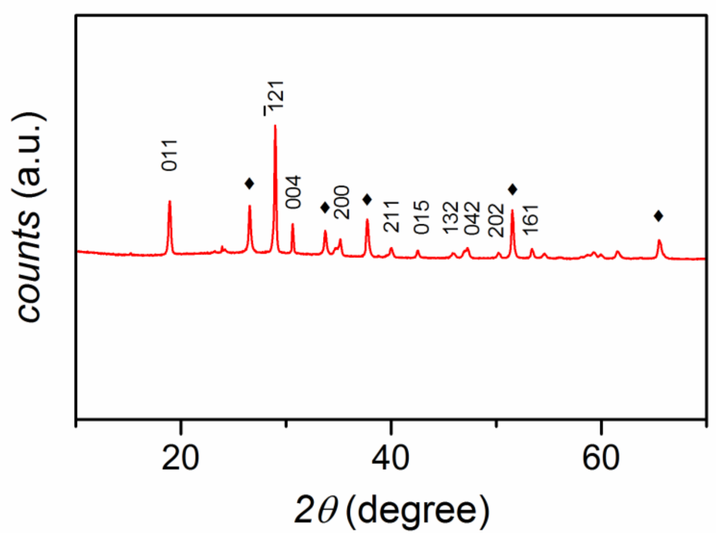

Supplementary Figure 1. Powder XRD diffrractrogram of a $BiVO_4$ film on FTO. The index numbers correspond to different lattice planes of $BiVO_4$. Diffraction attributed to FTO are indicated by black diamonds.

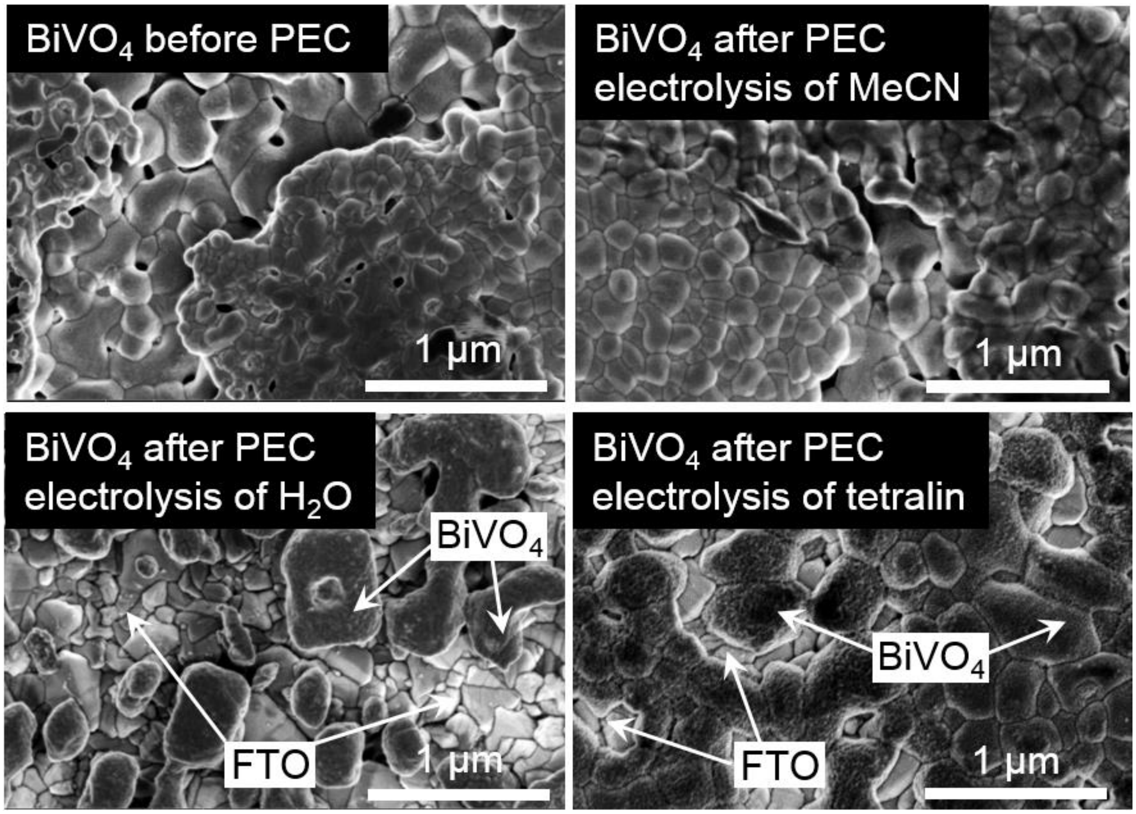

Supplementary Figure 2. SEM images before and after 96 h PEC electrolysis of H_2O, MeCN or tetralin show full surface coverage of $BiVO_4$ on FTO before PEC and after PEC electrolysis of MeCN, and more surface corrosion of $BiVO_4$ after PEC electrolysis of H_2O relative to tetralin.

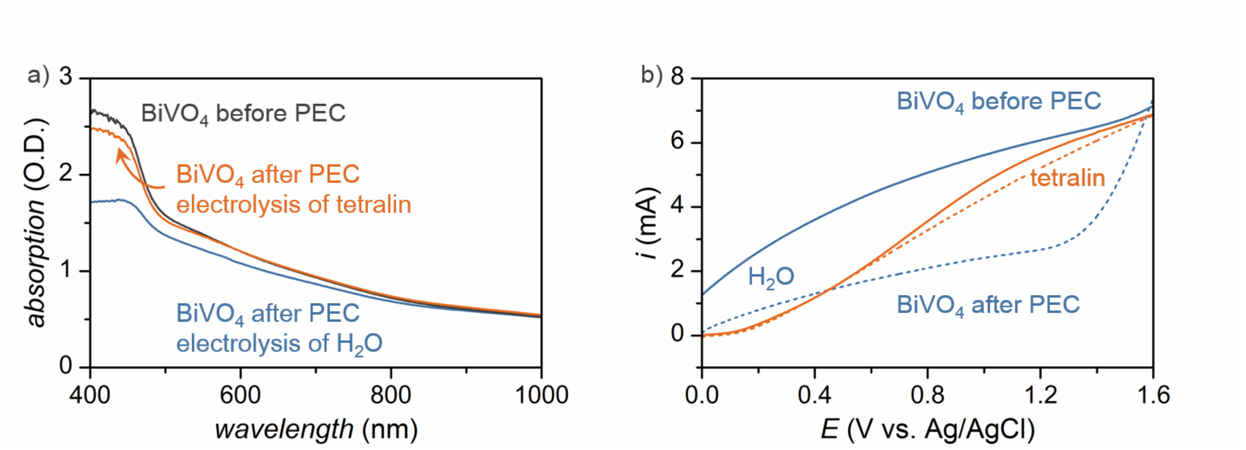

Supplementary Figure 3. (a) UV-vis absorption spectra for $BiVO_4$ photoanodes before (black) and after 96 h of PEC electrolysis of tetralin (orange) or H_2O (blue). (b) Photocurrents of $BiVO_4$ photoanodes before (solid line) and after 96 h (dashed line) of PEC electrolysis of tetralin (orange) or H_2O (blue).

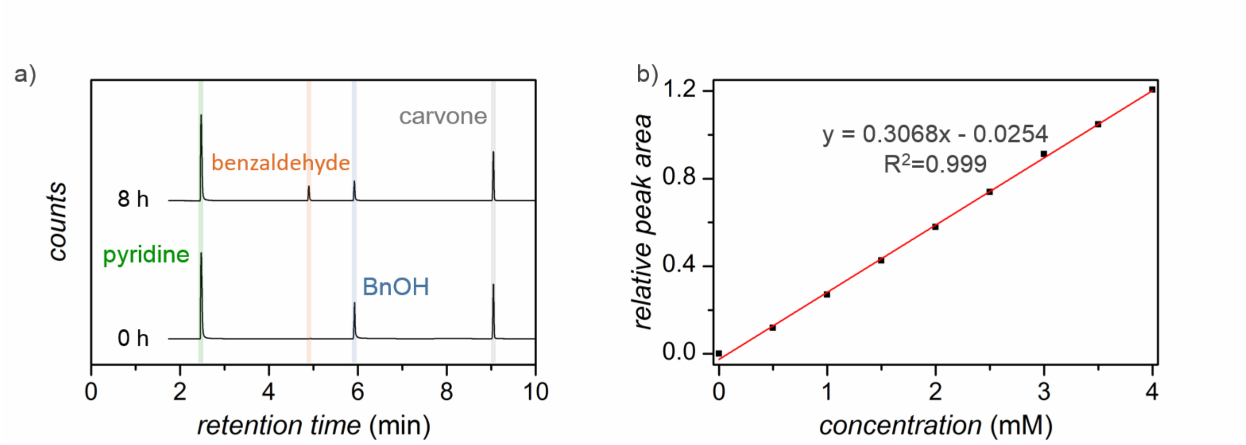

Supplementary Figure 4. (a) GC-MS traces of PEC oxidation of benzyl alcohol (BnOH) into benzaldehyde. (b) Calibration curve of benzaldehyde made by plotting the relative peak area versus concentration of benzaldehyde. (Carvone was used as the internal standard.)

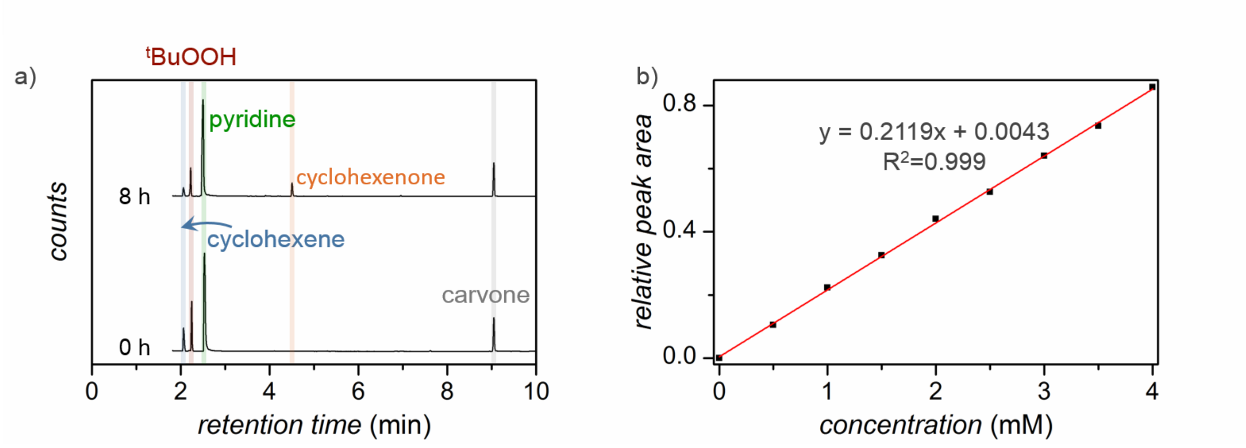

Supplementary Figure 5. (a) GC-MS traces of PEC oxidation of cyclohexene into cyclohexenone. (b) Calibration curve of cyclohexenone made by plotting the relative peak area versus concentration of cyclohexenone. (Carvone was used as the internal standard.)

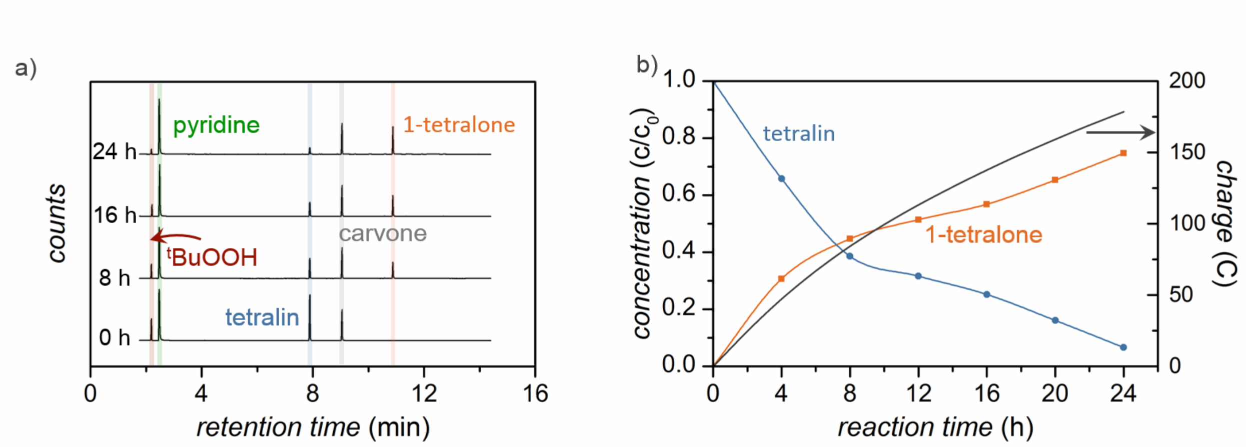

Supplementary Figure 6. (a) GC-MS traces of PEC oxidation of tetralin into 1-tetralone. Carvone was used as the internal standard. (b) Relative concentrations of tetralin (blue) and 1-tetralone (orange), and charges passed through the PEC cell (black) during the PEC oxidation of tetralin. Concentrations were quantified by internal standard calibration on GC-MS.

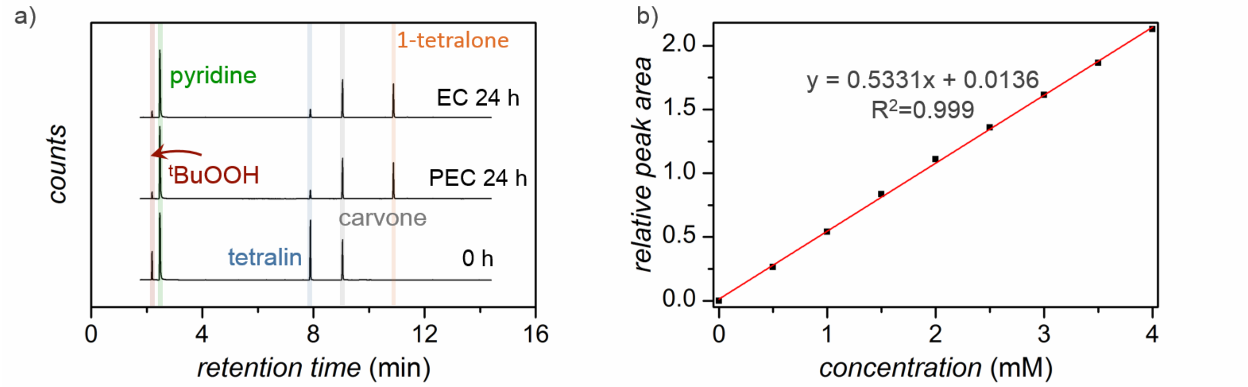

Supplementary Figure 7. (a) GC-MS traces of PEC and EC oxidations of tetralin into 1-tetralone. (b) Calibration curve of 1-tetralone made by plotting the relative peak area of 1-tetralone versus concentration of 1-tetralone. (Carvone was used as the internal standard.)

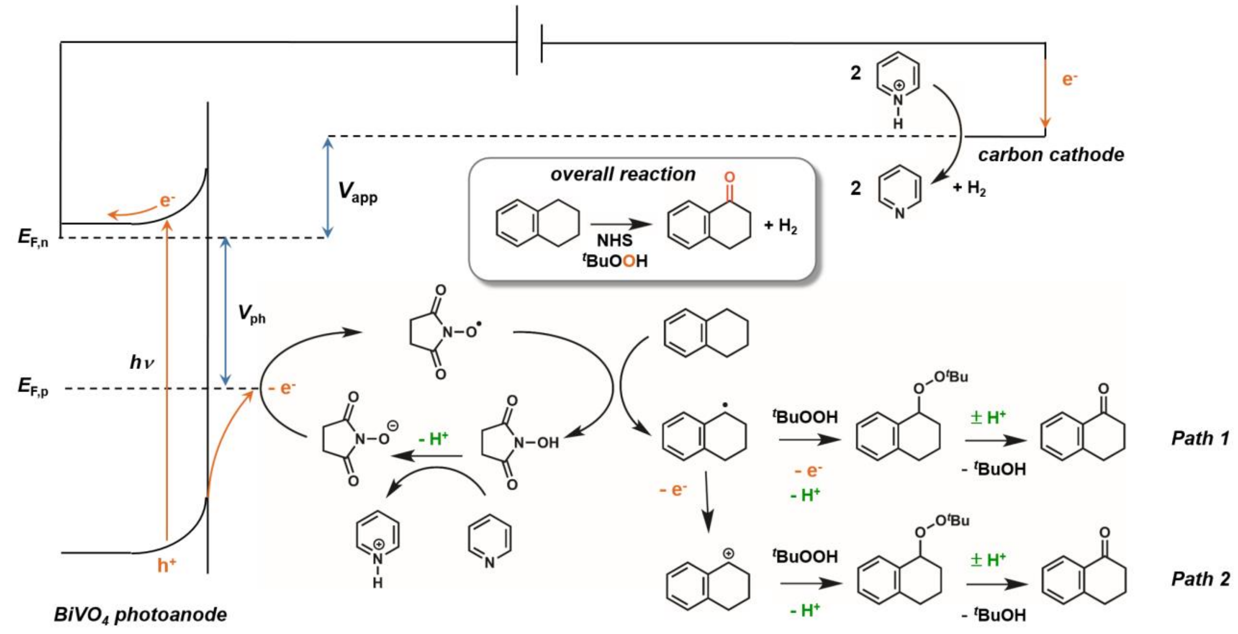

Supplementary Figure 8. Proposed mechanism of PEC oxidation of tetralin in MeCN. $E_{F,n}$ and $E_{F,p}$ = quasi-Fermi levels of photo-generated electrons and holes, respectively; V_{ph} = photovoltage; V_{app} = external voltage applied. It is likely that the reaction proceeds through a cationic intermediate (pathway 2), however, we cannot rule out the possibility of a pure radical mechanism (pathway 1) for the oxidation process.

Supplementary Table 1. Relative decay of photocurrents (measured at 1.2 V vs Ag/AgCl) of $BiVO_4$ after 96 h of PEC electrolysis in a mixture of H_2O and MeCN.

H_2O:MeCN	relative decay of photocurrent
0:10	7%
1:9	18%
2:8	21%
3:7	26%
10:0	56%
